# A Whiff of Danger: Synthetic Musks May Encourage Toxic Bioaccumulation

**Published:** 2005-01

**Authors:** Cynthia Washam

A class of widely used fragrances that are considered nontoxic may pose a hidden threat to human health by enhancing the effects of compounds that *are* toxic—a paradox discovered by Stanford University researchers Till Luckenbach and David Epel in a recent study of synthetic musk compounds **[*EHP* 113:17–24]**. The duo, based at Stanford’s Hopkins Marine Station, found that musks inhibited natural defenses against toxicants in California mussels, and that the effect remained long after exposure. Their findings raise a red flag for human health because musk compounds concentrate in fats (including breast milk) and endure in human tissue long after exposure.

People typically are exposed to musks transdermally, through soap, cosmetics, and clothes washed with scented detergents. Musks also are inhaled, through cologne sprays. Every year, some 8,000 metric tons of the inexpensive synthetic fragrances are produced worldwide.

The discovery of musk compounds in human fat a decade ago prompted Japan and Germany to ban some musk compounds. German researchers who measured human body burdens found musks in the fat of all their subjects and concluded that humans are constantly exposed to these highly stable compounds. The United States and other countries, though, allowed continued use of the fragrances because they were considered safe; a battery of routine toxicology screens have shown musk compounds to be nontoxic.

Epel and Luckenbach speculated that musks enhance the effects of toxicants by confounding cellular defense systems. Cells naturally resist toxicants through multidrug/multixenobiotic resistance (MDR/MXR) efflux transporters, proteins that keep foreign chemicals from entering cells. Epel and Luckenbach built on earlier findings reported in the September 1997 issue of *EHP Supplements* that man-made fat-soluble chemicals could inhibit MDR/MXR efflux transporters. Because musks are fat-soluble, they suspected synthethic musk compounds of having this effect.

The researchers chose mussel gill tissue for their study because its efflux transporters are particularly active. They incubated the tissue for 90 minutes in a solution containing musk compounds and the fluorescent dye rhodamine B. The dye reflects efflux transporter activity; finding rhodamine B in the tissue would indicate the transporters were failing.

Immediately after incubation, Epel and Luckenbach found rhodamine B uptake to be 38–84% higher in tissue treated with musk compounds than in controls. They were surprised to find, 24 hours later, that rhodamine uptake was still 30–74% higher in tissue exposed to musks. Efflux transport remained compromised 48 hours after exposure in tissue treated with certain commonly used compounds: musk xylene, musk ketone, Galaxolide, and Celestolide. Only tissue exposed to the compounds Traseolide and Tonalide recovered before 48 hours postexposure.

Epel and Luckenbach believe their study is the first to demonstrate long-term inhibition of the MDR/MXR system by synthetic musks. They warn that musk compounds, and possibly other chemicals as well, might similarly compromise the MDR/MXR system in humans. Evidence for this theory comes from the effectiveness of chemosensitizing drugs, which inhibit efflux transporters much as musk compounds do. Chemosensitizers are now being tested in clinical trials to prevent tumor cells from resisting harsh chemotherapeutics.

Luckenbach and Epel conclude that it is important to determine whether musks and other chemicals cause similar effects in humans. If so, they write, the result could be unanticipated accumulation of toxicants that would confound safety predictions of seemingly harmless chemicals.

## Figures and Tables

**Figure:**
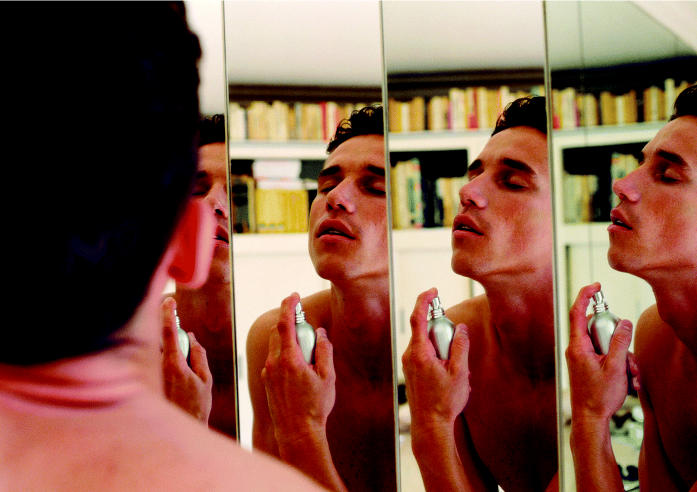
**The science of scents.** New data on musks show the compounds may inhibit cellular defenses against chemicals and bioaccumulate, with potentially hazardous results.

